# Episodes, events, and models

**DOI:** 10.3389/fnhum.2015.00590

**Published:** 2015-10-27

**Authors:** Sangeet S. Khemlani, Anthony M. Harrison, J. Gregory Trafton

**Affiliations:** Naval Research Laboratory, Navy Center for Applied Research in Artificial IntelligenceWashington, DC, USA

**Keywords:** event segmentation, temporal reasoning, mental models, episodic memory, MDS robot, ACT-R/E

## Abstract

We describe a novel computational theory of how individuals segment perceptual information into representations of events. The theory is inspired by recent findings in the cognitive science and cognitive neuroscience of event segmentation. In line with recent theories, it holds that online event segmentation is automatic, and that event segmentation yields mental simulations of events. But it posits two novel principles as well: first, discrete episodic markers track perceptual and conceptual changes, and can be retrieved to construct event models. Second, the process of retrieving and reconstructing those episodic markers is constrained and prioritized. We describe a computational implementation of the theory, as well as a robotic extension of the theory that demonstrates the processes of online event segmentation and event model construction. The theory is the first unified computational account of event segmentation and temporal inference. We conclude by demonstrating now neuroimaging data can constrain and inspire the construction of process-level theories of human reasoning.

## Introduction

How do people represent and reason about time? Calendars, clocks, and timepieces come coupled with the convenient illusion of time as a collection of discrete temporal markers, such as months and minutes, which are experienced in serial order. Events, such as *breakfast* or *the birthday party*, are perceived as hierarchical organized structures relative to those markers. In extraordinary conditions of sensory deprivation—a prisoner in solitary confinement, for example –the façade of a regimented temporal hierarchy melts away to reveal the truth: time at the scale of human experience is a continuous flow of sensory information without subdivision.

Humans organize this unabating stream of sensory input into meaningful representations of episodes and events. Brain regions are sensitive to perceptually salient event boundaries (Zacks et al., 2001a), and people learn to segment continuous actions into discrete events in their infancy (Wynn, 1996). The concept of time, temporal order, and event structure develops throughout childhood (Piaget, 1927/1969; Harner, 1975; Hudson and Shapiro, 1991). By age 3, children understand the temporal order of actions and their relations to one another in a sequence of conceptually related events (Nelson and Gruendel, 1986). Adults in turn rely on complex event structures in comprehending discourse and temporal expressions (Miller and Johnson-Laird, 1976; Moens and Steedman, 1988), in remembering autobiographical episodes (Anderson and Conway, 1993), and in planning for the future (Bower, 1982). The end result of parsing the continuous stream of sensory information appears to yield event structures that take the form of a mental model, i.e., an iconic configuration of events organized around a spatial axis (Johnson-Laird, 1983; Casasanto et al., 2010; Radvansky and Zacks, 2011; Bonato et al., 2012), from which temporal relations between can be inferred (Vandierendonck and De Vooght, 1994; Schaeken et al., 1996; Gentner, 2001).

There is an intimate link between the processes of temporal inference and the way in which the brain segments events: event segmentation yields the mental representations that permit temporal reasoning. Recent research focuses on how the brain carves continuous experiences up to build discrete temporal representations. Behavioral and imaging data suggest that to construct representations of events online, individuals rapidly integrate multiple conceptual and perceptual cues—such as a movement to a new spatial location or the introduction of a new character or object into the perceiver's environment (Zacks et al., 2007). But no theory describes how cues are accessed and encoded, how they are integrated, and how they are used to build representations of events; no extant computer program can solve the task either.

To address the discrepancy, we describe a novel approach that synthesizes these various operations to yield a unified theory of event segmentation and temporal inference. We implemented the system computationally in an embodied platform that is able to process input from its sensors to build discrete model-based representations of events. The paper begins with a review of the functional neuroanatomy of the brain mechanisms underlying the integration of conceptual and perceptual cues to mark event boundaries. It then describes a theory of how processing continuous sensory information yields episodic memory representations, as well as how those memory representations are used to build event models. It presents a computational and robotic implementation of the theory, and shows how the theory provides a foundation for an account of temporal inference. Finally, it reviews the present approach as one that marshals the insights of cognitive neuroscience to advance theories of high-level inference.

## Event segmentation in the brain

You walk through a hallway to enter a room, where your colleague sits behind her desk. You take a seat in front of the desk and begin to converse with her. You leave the office sometime later to head to the bar across to street to meet a friend for drinks. At some point during this sequence of continuous environmental changes, a new event began: the *meeting*. At another point, it ended and a new event began. There exists no direct, observable, physical cue that marks the beginning, duration, or end of the meeting: the meeting and its extension across time has to be perceived indirectly from an integration of multiple internal and external cues (Zacks and Tversky, 2001), and the process of perception has to yield a discrete representation of a sequence of events (Radvansky and Zacks, 2011).

People can systematically parse out meaningful events by observing sequences of everyday actions (Newtson, 1973; Newtson et al., 1977). Newtson and his colleagues pioneered the study of event segmentation behavior, and posited three hypotheses on the perception of events: first, event boundaries are distinguished by a large number of distinctive changes in perceptual stimuli. Second, event boundaries are graded—some boundaries are sharp and mark distinct separations between two separate events, whereas other boundaries are fuzzier and mark less distinguished separations. Finally, events are part of a “partonomy,” i.e., a part-whole hierarchy (see Cooper and Shallice, 2006; Hard et al., 2006). For example, suppose you *wash a set of dirty dishes*. That event consists of subordinate events (e.g., *wash plate 1, wash plate 2*, and so on) and is itself part of a larger event (e.g., *cleaning the kitchen*).

Recent neuroimaging studies concur with Newtson's proposals. Zacks and his colleagues present decisive evidence that processes governing event segmentation are unconscious, automatic, and ongoing (Zacks et al., 2001a, 2010; Speer et al., 2003). In one study, participants passively viewed sequences of everyday activities in the scanner, and then viewed the sequences again while they explicitly segmented the event boundaries (Zacks et al., 2001a). The data revealed systematic increases in BOLD response prior to points at which boundaries were identified; likewise, there was a reliable difference in activation of frontal and posterior clusters of brain regions as a function of whether participants marked fine or course boundaries in events. These two points suggest an ongoing, automatic segmentation process that integrates cues from external stimuli in the absence of conscious deliberation. A similar study by Speer et al. (2003) revealed that evoked responses in the brain's motion sensitive area (extrastriate MT+ and the area connecting left inferior frontal and precentral sulcus) occurred in temporal proximity to participants' overt segmentation behavior as they analyzed videos of action sequences. Schubotz and colleagues show that MT activation may play a more general role in segmenting ongoing activity from movements, i.e., not just for goal-directed action sequences (Schubotz et al., 2012). Participants' behavioral data likewise provide evidence for partonomic organization of event segmentation: their subjective evaluations of coarse event boundaries overlap with their evaluations of fine boundaries (see Zacks et al., 2001a). Moreover, when asked to describe events from memory, participants' responses reveal a hierarchical structure such that superordinate events are remembered and described more frequently (Zacks et al., 2001b).

Online event segmentation is not driven by visual cues alone. Speer et al. (2009) found an association between activations in regions of the brain associated with processing event boundaries and participants' identification of event boundaries in linguistic narratives. Event boundaries were distinguished by explicit changes in characters, locations, goal-directed activities, causal antecedents, and interactions with objects in the narratives (Speer et al., 2009). Other evidence reveals brain regions that subserve online event segmentation in auditory narrative comprehension (Whitney et al., 2009) and in music (Sridharan et al., 2007).

These results dovetail with other work that suggests that understanding action narratives is similar to simulating motor movements (e.g., Aziz-Zadeh et al., 2006). Aziz-Zadeh et al. show that mirror neuron areas in the premotor cortex are active both when participants passively observe action sequences as well as when they read descriptions of those same sequences. As they argue, the results support the activation of shared mental representations for conceptually interpreting language input and for perceptually processing visual input.

In sum, neural evidence corroborates three hypotheses about event segmentation:

1. Event segmentation is an ongoing, automatic process.
2. Events are segmented into discrete representations relative to a temporal partonomy, where events are embedded within other events. An additional computational constraint is that because the brain cannot represent infinite regression, the temporal partonomy must be bounded.
3. Event segmentation is driven by detecting perceptual changes in audiovisual stimuli and in conceptual changes in mental representations of discourse (but cf. Schapiro et al., 2013).

### Gaps in theories of event perception

It may be unimpeachable that people systematically carve continuous experience into events, and that they do so by marking boundaries between events. Many views from philosophy, neuroscience, and psychology even concur that event structures are discrete in nature (e.g., Casati and Varzi, 2008; Radvansky and Zacks, 2011; Liverence and Scholl, 2012) and some theorists posit specific ways in which those structures can be organized relative to one another (Schapiro et al., 2013). Indeed, few would argue that representations of event structure aren't critical for making inferences about temporal, spatial, and causal relations. However, consensus over matters of event cognition does not imply completeness. No extant theory of event segmentation explains how the process yields discrete event representations. Instead, many gaps in knowledge exist about how event structures come about. Three salient questions remain unanswered by theoretical and empirical investigations: First, what is the neurocognitive representation of an event boundary? It may be a discrete representation that is encoded in memory, or it may be a transient set of activations that are rapidly extinguished once a representation of an event is constructed. Second, how does the online process of event segmentation resolve multiple perceptual and conceptual segmentation cues? Some cues appear more important than others, e.g., changes in the focus of an object may be less important than changes in location, and other cues may compete with one another. Third, how does the brain recognize an event as an event? In addition to encoding an event's spatiotemporal frame, its characters, their goals, their interactions, and the objects involved, the mind needs to represent a nested structure of events within other events, and no theory at present explains what the representation looks like or what sorts of mental operations are permitted by it.

To address these three questions, we developed a novel theory of event segmentation and temporal inference. The theory builds on the idea that changes to internal and external stimuli precipitate segmentation behavior, but goes beyond it to hypothesize that segmentation is driven by the construction of episodic representations of event boundaries. Some perceptual and conceptual cues take precedence to others to yield a precedence hierarchy, and the hierarchy determines the activations of episodic representations in memory. The episodic memories in turn allow for the direct construction of mental models of temporal relations. We present the theory in the next section.

## A unified theory of event segmentation and representation

We developed a novel, model-based theory of event segmentation and event representation. The theory inverts a common strategy in understanding event segmentation: instead of considering how individuals parse a continuous stream of information into discrete temporal units, we begin with the assumption that the end result of segmentation is the construction of a temporal mental model (Johnson-Laird, 1983; Schaeken et al., 1996; Radvansky and Zacks, 2011). Craik (1943) was the first psychologist to propose that people build and interrogate small-scale models of the world around them, but philosophers before him explored analogous notions. Mental models serve as a general account of how individuals perceive the external world, how they understand linguistic assertions, how they represent them, and how they reason from them (see Johnson-Laird, 1983; Johnson-Laird and Byrne, 1991; Johnson-Laird and Khemlani, 2014). As Johnson-Laird (1983, p. 406) writes, “Mental models owe their origin to the evolution of perceptual ability in organisms with nervous systems. Indeed, perception provides us with our richest model of the world.” Hence, models serve as a way to unify perceptual and linguistic processes, as they are hypothesized to be the end result of both. They are pertinent to reasoning about abstract relations, as well as relations about time and space (Goodwin and Johnson-Laird, 2005; Ragni and Knauff, 2013). The model theory depends on three foundational principles:

1. Mental models represent distinct *possibilities*: when perceiving the world and processing language, models represent a set of discrete possibilities to which the current situation or description refers. When perceiving the world, models represent a homomorphism of the sensory input, i.e., many properties of the sensory input are omitted from the model. The properties that are represented are subject to the next principle of the theory.
2. The principle of *iconicity:* a model's structure corresponds to the structure of what it represents (see Peirce, 1931–1958, Vol. 4). Events are represented as either kinematic models that unfold in time, i.e., where time is represented by time itself akin to a mental “movie” (Khemlani et al., 2013) or else as a spatial arrangement of discrete events, where time is represented along a mental time line (Schaeken et al., 1996; Bonato et al., 2012). Logical consequences emerge from the iconic properties of the models (Goodwin and Johnson-Laird, 2005) and conceptual simulations on the models (Trickett and Trafton, 2007; Khemlani et al., 2013).
3. The principle of *parsimony*: In scenarios in which discourse is consistent with multiple alternative models, people tend to construct a single mental model, which yields rapid, intuitive inferences. Provided that the inferential task is not too difficult, they may be able to construct additional alternative models from a description. However, inferences that depend on alternative models are more difficult.

Mental models account for how people reason about time. Schaeken et al. (1996) showed that reasoners are faster and make fewer errors when reasoning about descriptions consistent with just one event model than descriptions consistent with multiple models. For example, the following description is consistent with one model:

John takes a shower before he drinks coffee.
John drinks coffee before he eats breakfast.

The event model consistent with premises can be depicted in the following diagram:

shower    coffee    breakfast

The diagram uses linguistic tokens arranged across spatial axis that represents a mental timeline. The tokens are for convenience, but the theory postulates that people simulate the events corresponding to each token. They make inferences by scanning the iconic representation for relations. When a token is to the left of a second token on the timeline, the event to which it refers happens before the event in the second token. Hence, reasoners have little difficulty deducing that John takes a shower *before* eats breakfast from the description. They do so rapidly and make few mistakes. In contrast, the following description is consistent with multiple models:

John takes a shower before he drinks coffee.
John drinks coffee before he eats breakfast.

The premises are consistent with the possibility in which the coffee precedes the breakfast:

shower    coffee    breakfast

and also with the possibility in which the breakfast precedes the coffee:

shower    breakfast    coffee

Reasoners have difficulty in deducing that no relation holds of necessity between the shower, the coffee, and the breakfast. They appear to build one model of the assertions and to refrain from considering alternatives (see also Vandierendonck and De Vooght, 1994, 1997). Vandierendonck and colleagues further showed that reasoners construct initial event models relative to their background beliefs (Dierckx et al., 2004).

The model theory accordingly serves as a viable account of temporal representation and reasoning, though the theory does not explain how events are perceived in the first place. In the following sections, we posit two novel assumptions that augment previous model-based accounts. The resulting theory can cope with how people represent durations, and also how they perceive durational events online. It accordingly provides a unified account of temporal perception and inference.

### Representing duration with models

One fundamental challenge to the theory presented above is that it does not account for how people represent and reason about events with durations. People make inferences about durations on a routine basis: if you are scheduled to take part in a meeting from 10 a.m. to 1 p.m., and a colleague asks you to join him for lunch at 12 p.m., then you must first detect the conflict and then prioritize your schedule accordingly. Hence, reasoners base their actions on understanding durations of events. While previous incarnations of the model theory have focused on punctate and not durational events, we extend the theory to deal with both. The reason is because many events can be construed in a punctual aspect, i.e., as taking place in a single moment, as well as in a durational aspect, i.e., one that describes a scenario that endures across a temporal interval (Miller and Johnson-Laird, 1976; Moens and Steedman, 1988). Consider the following examples from Miller and Johnson-Laird (1976, p. 429–431):

- It exploded when he arrived.
- It exploded while he arrived.

In (a), the sentential connective *when* ensures that the noun phrase, *he arrived*, takes on a punctual aspect. Hence, people may build a model akin to the following:

arrived
exploded

where the two events happen at same time and are therefore vertically aligned (given a horizontal axis representing time). In (b), the connective *while* confers a durational aspect, and so people may directly represent the duration in their mental model, e.g.:

[   arrived   ]
exploded

where the brackets denote that the arrival is extended across several time points. As both punctate and durational events are pervasive in daily life, a rich account of temporal reasoning must explain how both types of events are represented and interrogated.

Durational events play an essential role in event perception. Events are almost always perceived across a temporal interval. If, as most theories of segmentation posit, people use environmental changes to mark the beginnings and endings of events, then events must extend across multiple moments in time for those changes to be registered. It may be that events are perceived at first as being durational in nature, and coalesce later into punctate moments only after being encoded in memory. Exceptions exist: the moment of birth, the moment of death, and winning the lottery may be perceived as a single moment in time. But many events are compiled into punctate representations only under retrospective analysis. The process of segmenting events assumes that segmentation is necessary to begin with, and hence, that most events subject to direct perception have duration.

An initial step to a unified theory of event segmentation and temporal inference is accordingly to explain how durations are represented in models. Models concern discrete possibilities; the theory eschews the representation of infinite sequences, and so metric information is difficult to represent with models of possibilities. One challenge is accordingly to describe a method by which durations are represented discretely. Recent work in cognitive neuroscience may provide insight into the nature of the representation. Research on rats reveals specific hippocampal neurons that fire reliably at particular moments in event sequences. These so-called “time cells” encode the event for later retrieval, as well as episodic information such as where the event takes place (MacDonald et al., 2011). Studies on adults corroborate the essential role of the hippocampus in encoding event sequences, encoding episodic information, and bridging temporal gaps between discontiguous events (Kumaran and Maguire, 2006; Lehn et al., 2009; Ross et al., 2009; Staresina and Davachi, 2009; Hales and Brewer, 2010). Ezzyat and Davachi (2011) show that event boundaries are used to bind episodic information to event representations; more generally, they posit a critical role of episodic memory in event perception. In a similar vein, Baguley and Payne (2000) present evidence that people encode episodic traces in memory, and use those traces to build event models from temporal descriptions.

We accordingly introduce the following principle about the representation of durations:

*The principle of discrete episodes:* Reasoners represent durational events by constructing discrete episode markers as chunks in episodic memory. Episode markers represent perceived changes in goals, locations, individuals, and objects. Markers are retrieved to construct durational mental models in which one marker represents the start of an event and another marker represents its end.

The principle of discrete episodes has implications for both event segmentation and mental model construction. According to the principle, when an event boundary is identified during online event segmentation, an episode marker is constructed. The event boundary may be triggered by multiple perceptual or conceptual cues; those cues are encoded in the representation of the marker (cf. Ezzyat and Davachi, 2011). For example, consider the scenario introduced in Section Event Segmentation in the Brain of a meeting with your colleague. The meeting might begin when you enter your colleague's office. Many changes occur the moment you enter: a change in location, the introduction of a salient individual to the environment (your colleague), the start of a goal (holding the meeting), and the introduction of a salient object (e.g., a printout of data). A single episodic marker encodes all of the detected changes: the location, the individual, the goal, and the object. When the meeting ends and you leave the office, there is a change in location, which may precipitate the construction of another episodic marker. Other things may or may not change; for example, if your colleague walks with you back to your office with the printout in hand, no character- or object-based changes would be encoded.

The principle posits that episodic markers are encoded as chunks in episodic memory (Altmann and Trafton, 2002, p. 40). As such, they are highly active when they are first constructed, but memory for them gradually fades. Markers that encode many perceptual and conceptual changes start with higher activations than markers that track fewer changes. Episodic markers are maintained in long-term memory (cf. Baguley and Payne, 2000), and when they are retrieved, their activation spikes and spreads to activate associated markers, i.e., those within the same temporal context and those that track the same sorts of perceptual and conceptual changes.

Episodic markers, by definition, encode punctate episodes. They can also be used retrospectively to construct discrete representations of events, i.e., durational event models. A memory of “the meeting” would accordingly consist of two separate markers as follows:

meeting_START_    meeting_END_

The markers may encode disparate sets of information. The start and end of a meeting may be cued by perceptual changes in location, for example, whereas the start and end of a bike ride concerns the conceptual introduction and completion of a goal (We address this issue in a thoroughgoing way in the next section). In either case, episodic markers can be used to build event models. Such models can be hierarchically organized:

**Table:** 

day_START_ day_END_
meeting_START_ meeting_END_
evening_START_ evening_END_
dinner
1 2 3 4 5 6 7

In the model above, each line represents a distinct event. The model depicts a punctate event (dinner) represented within a durational event (the evening). The dinner may be conceived as durational as well, but at the bottom of the hierarchy, non-intersecting durational events are functionally equivalent to punctate events. The model is iconic and its components are discrete, i.e., it does not maintain any metric information by default, such as how many minutes the “day” event endured or how many hours the “morning” event endured; hence, people can reason about events whose durations outlast lifetimes (e.g., epochs and eons). Humans and other animals use other neural mechanisms to track and represent metric information about duration (see Allman et al., 2014, for a review). The numbers represent individual episode markers, e.g., 3 represents the episode marker that encodes the cues used to mark the end of the meeting. It is also a parsimonious representation from which to make temporal inferences. For example, the model above can be used to infer the following temporal relations:

- The dinner did not occur during the meeting.
- The meeting occurred before the evening.
- The dinner happened during the day.

Hence, relations concerning relative duration and other temporal relations can be drawn from models that maintain only discrete representations. The principle of discrete episodes posits that episode markers are used to construct events dynamically and to retrospectively build representations of events from memory or linguistic descriptions.

### Constructing models dynamically from episodic information

According to the principle of discrete episodes, episode markers encode perceived changes in goals, locations, and other salient conceptual and perceptual information. But how can the system use the information encoded within an episode marker to rapidly construct event models dynamically, even as new markers are being encoded? The problem is acute because the cues used to mark the beginning of an event may not be relevant in marking the end of an event. The process of interrogating all of the information encoded by an episodic marker is cognitively implausible on account of the combinatorial explosion inherent in assessing and integrating multiple types of properties. The theory accordingly posits a more rapid procedure:

*The principle of event prioritization:* Events are associated with a single perceptual or conceptual element whose change denotes the beginning and end of the event. Changes in elements are prioritized with respect to a given context: by default, goal events are the highest priority as they override events based on perceptual changes. When a goal is active, perceptual changes do not yield episode markers outside the context of the goal. Perceptual changes are likewise ranked in order of priority based on the ease of detecting a change: location events override events based on individuals, which in turn override those based on objects in the environment.

One way of construing the principle of event prioritization is that an ongoing event completes only when elements of the highest pertinent priority change. Recent work uncovers evidence for the prioritization and ordering of rule sets (Reverberi et al., 2012), and we extend the general idea to focus on event perception. In what follows, we describe how the principle operates for four primary sorts of conceptual and environmental changes: goals, locations, individuals, and objects.

#### Goals

The principle posits that goal-directed events are of utmost importance. Here we speak of goals in a narrow sense: goals are mental states that govern immediate, short-term, and ongoing sequences of actions that bring about a desired state of affairs in the world. Hence, goal-directed actions are those that subserve the completion of the goal. Life goals, career goals, and romantic goals are outside the scope of our present analysis because they do not govern immediate, short-term sequences. Many seminal studies on event representations address the integral involvement of goals in the way events are encoded, retrieved, and reconstructed (Lichtenstein and Brewer, 1980; Brewer and Dupree, 1983; Travis, 1997). Goals are of highest importance because they provide a top-down structure on event segmentation based on perceptual changes. An example of a sort of goal that falls within the purview of the principle of event prioritization is the goal to walk across town to meet a friend for a drink at a prearranged time. The goal-based event (walking across town) continues until the goal is completed. While episodic markers are constructed as the event proceeds, the perceived event remains organized relative to the goal and not on any other perceptual experience, such as the perception of changes in locations or individuals in the environment. Hence, external cues that would otherwise signal the beginning of a new event—such as a change in location—would instead signal the beginning of a new subevent organized within the context of the goal-based event.

#### Locations

Locations serve to organize multiple perceptual stimuli. As with the time cells discussed above, animals and people have dedicated hippocampal “place cells” that encode location information (see Moser et al., 2008, for a review). A behavioral demonstration of their importance is evident in studies by Radvansky and Copeland (2006) and Radvansky et al. (2010). They show that memory for objects drops when individuals move through a doorway from one location to another in a virtual reality environment, and explain the effect as a dynamic update to an event model. The principle of event prioritization posits that locations govern the perception of an event when a high-level goal stays constant and ongoing, or is absent altogether. Locations are also more stable than other sorts of perceptual stimuli because locations generally do not change relative to another individual's agency, whereas other sorts of perceptual cues (the individuals in the environment and the objects they interact with) do change relative to agency. We discuss them next.

#### Characters and objects

Characters and objects in an environment serve as low-level perceptual cues for the dynamic construction of events in the absence of both goal- and location-based cues. When individuals have no goal to govern their actions and their locations do not change for a long period of time (e.g., when traveling on an airplane for several hours), the principle of event prioritization posits that dynamic events are constructed relative to detecting changes based on interaction, i.e., changes in individuals and changes in objects to which the perceived attends. One motivation for the deference of character- and object-based cues to goal- and location-based cues is that the former two can change rapidly, and it requires computational resources to track those changes and use them to update event models. Another motivation comes from evidence from Zacks et al. (2001b): they asked participants to describe units of activity as they identified them in an event segmentation task with instructions to mark events using a fine-grain or a coarse-grain. Participants described objects more often using fine-grain descriptions, and they used a broader variety of words to describe objects for fine-grained descriptions. These data suggest that people track objects more frequently when locations and goals do not change. The principle of event prioritization predicts that they may forget objects as locations change, in line with the results from Radvansky et al. (2010).

### Summary

**Figure 1:**
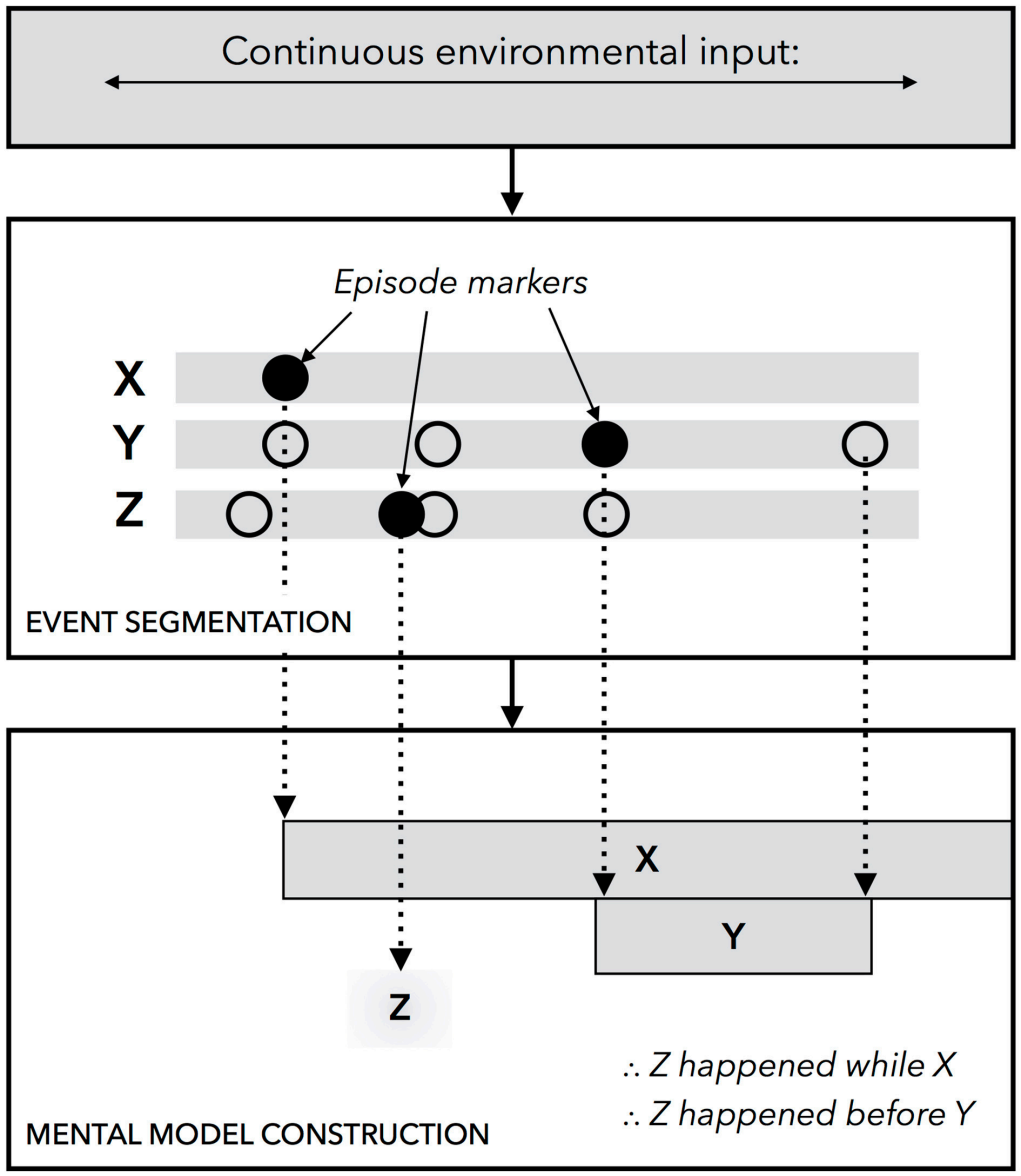
**A diagram of the unified theory of event segmentation and representation**. In the event segmentation component of the system, which operates online and in parallel with other cognitive processes, changes are detected in continuous environmental input across a finite set of perceptual stimuli, marked by X, Y, and Z in the diagram. At the onset of a stimulus, which is indicated by a black circle, a new episodic marker is constructed. The offset of a stimulus likewise yields a new episodic marker. When the system is queried for information pertaining to temporal relationships, it uses the markers to build a discrete event model. The system then scans the model to make inferences.

The unified theory of event segmentation and event representation that we posit is based on the assumption that segmentation yields and reasoning relies on mental models of temporal relations. Previous model-based accounts could not explain how durations were represented or how models were constructed dynamically, and so our unified account includes two novel assumptions: first, people track changes in their environment by automatically constructing discrete units of episodic memory, i.e., episode markers; and second, people dynamically construct events by prioritizing some cues over others. A summary of the theory is provided in Figure 1. To test the viability of the account, we turn next to describe its embodied computational implementation.

### An embodied implementation of the unified theory

**Figure 2:**
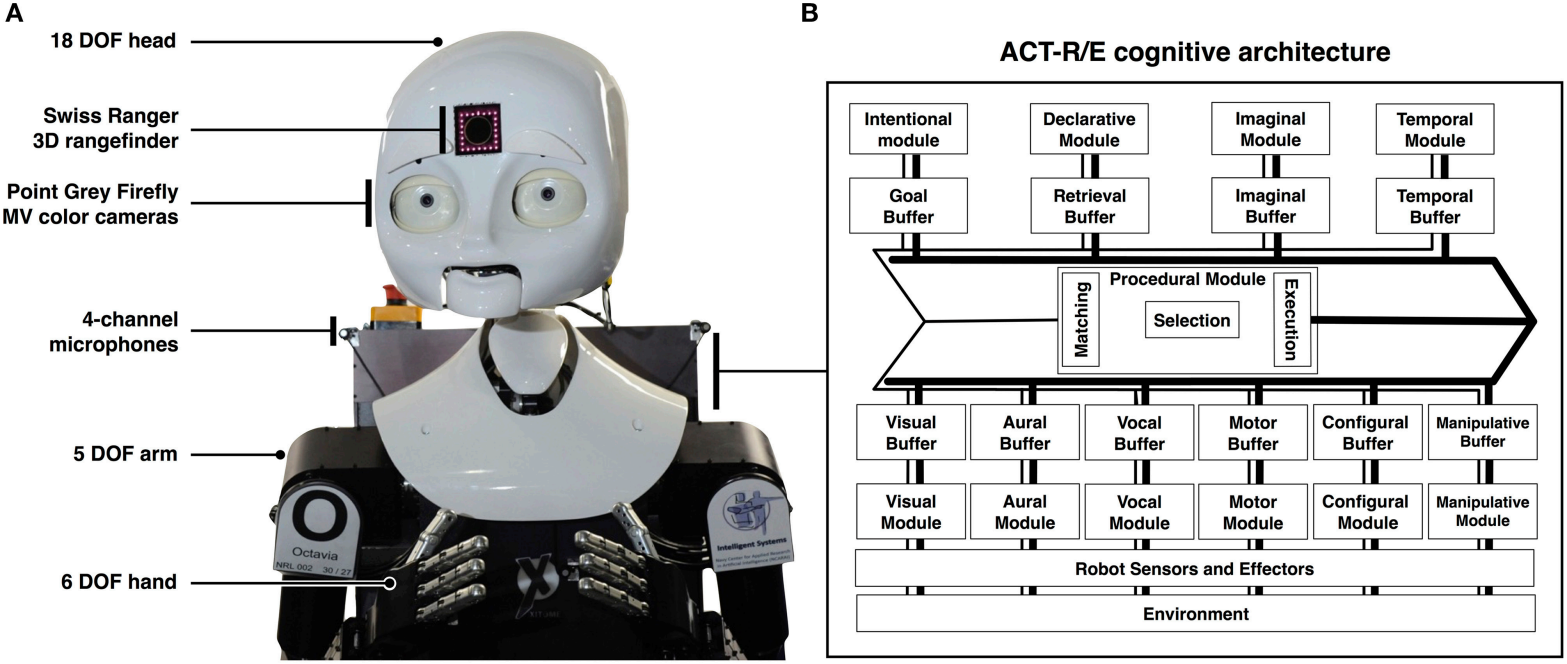
**The robotic implementation of the ACT-R/E cognitive**. **(A)** depicts the MDS (mobile, dexterous, social) robot in use in our lab, and shows its various sensors and effectors. **(B)** provides the details of the ACT-R/E cognitive architecture (Trafton et al., 2013). The architecture is an *embodied* extension of ACT-R (Anderson, 2007), and it interfaces the robot's sensory apparatus. ACT-R/E is composed of multiple modules that mimic components of human cognition. For example, it includes modules for maintaining goals, storing declarative memories, processing visual, and auditory input, and issuing motor commands. Each module is paired with a buffer that limits the capacity that the system can process at once, and accordingly implements a processing bottleneck characteristic of human cognition. Computational implementations of cognitive processes, such as the event segmentation system we present, are developed in ACT-R/E by constructing procedural memory representations that are executed under pre-specified conditions, and which retrieve information from or else modify the contents of the system's various buffers. In the diagram, the thin lines depict the pipeline for retrieval from the contents of the buffers and the thick lines depict the pipeline for modifying the contents of the buffers.

We developed an embodied, robotic implementation of the theory described in the previous section. The unorthodox approach is a result of the multifaceted nature of the tasks under investigation. The approach may be highly relevant for roboticists, because many robotic systems lack the ability to perceive and construct representations of events (Zacks, 2005; Maniadakis and Trahanias, 2011). But our goal is different. We argue that an embodied demonstration of the theory at work can help identify the types of information needed for the algorithms at each stage of the theory. A viable theory of event segmentation is one that integrates multiple perceptual and conceptual cognitive processes such as goal maintenance, location detection, person identification, and object recognition, and only a working system that integrates these perceptual processes sufficiently constrain and inform the implementational details of the theory we developed. Recent work in our laboratory has focused on each of these constituent perceptual processes: we have developed an embodied robotic platform capable of fiducial-based location tracking (see Kato and Billinghurst, 1999), person identification through face recognition (Kamgar-Parsi and Lawson, 2011) and soft biometrics (i.e., clothing, complexion, and height cues; Martinson et al., 2013) and context-sensitive object detection (Lawson et al., 2014). The platform's sensors and perceptual subsystems are interfaced with ACT-R/E, an embodied cognitive architecture for human-robot interaction (Trafton et al., 2013) based on ACT-R, a hybrid symbolic/subsymbolic production-based system for mental processing (Anderson, 2007). The system comes with multiple interoperating modules that are designed to deal with different sorts of inputs and memory representations called “chunks.” Modules make chunks available through a capacity-limited buffer. Modules and buffers are mapped to the functional operation of distinct cortical regions. ACT-R/E builds on the ACT-R theory in that it can parse environmental input from perceptual systems, which is translated into chunks in a long-term memory store (the “E” stands for “embodied”). ACT-R/E is also interfaced with robotic sensors and effectors, and so it can act on the physical world. A summary of the system's sensors and its cognitive architecture is provided in Figure 2. We briefly review how the system implements event segmentation and the construction of event models.

#### Online episodic segmentation

The principle of discrete episodes posits that at the lowest level, an agent's experience is carved up into discrete windows of time by the encoding of episodic markers. As an agent's goals, locations, and observations of objects and people change, new episodic markers are encoded and annotated with the type of change (e.g., a change in *location*) and the contents of the change (e.g., *entered location-b*). The markers do not represent temporal durations, but rather single points in time. Encoding happens automatically as a natural consequence of attending to the environment. In the ACT-R/E cognitive architecture (Trafton et al., 2013) when the computational implementation attends to a new goal, a representation of that goal is placed within the system's goal buffer. The system monitors the buffers of relevance (i.e., the *goal buffer* for goal changes, the *configural buffer* for location changes, and the *visual buffer* for people and objects; see Figure 2). It creates a new episodic marker when a change in content is detected (Altmann and Trafton, 2002; Trafton et al., 2011). Each episode is symbolically annotated with information regarding environmental changes. It is also associatively linked to the prior and new contents, as well as the prior episode marker. Linking the markers in this way permits subsequent retrievals to iterate through episodes and their associated contents.

**Figure 3:**
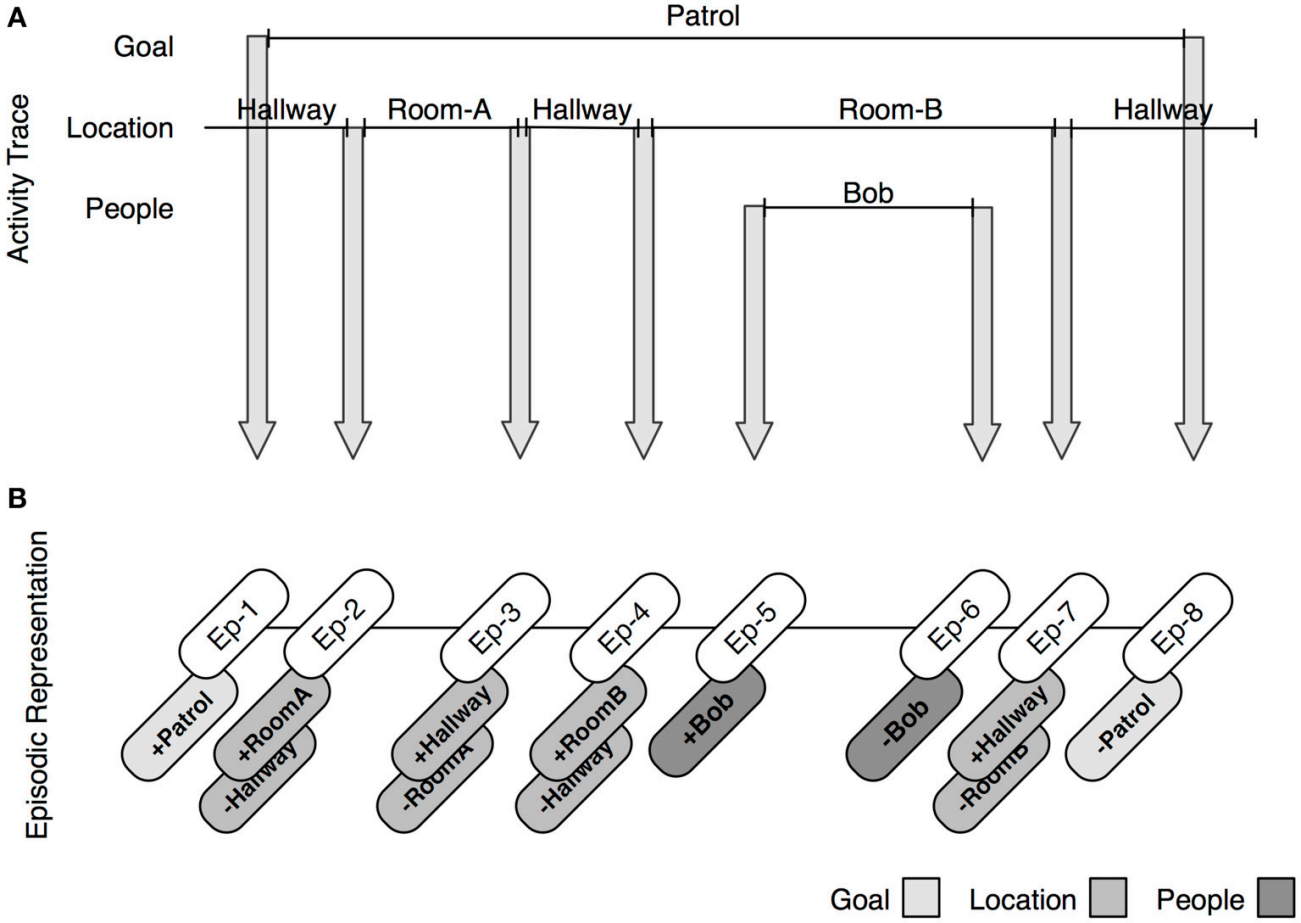
**The process by which episodes are encoded at event boundaries**. **(A)** Shows a diagram of a trace of activity as a function of changes in goals, locations, and people. At each change, a new episodic marker is constructed (depicted as arrows). **(B)** Shows the representation of each episodic marker. Episodes are linked with symbolic information that describes the perceived changes at the time of encoding. Hence, episodes are used to uniquely describe a change in goal, location, person, and object (not depicted).

Figure 3 provides a detailed trace of the creation of discrete episodic markers. At the top of the figure is an activity trace for an individual patrolling an area. When the goal of patrolling is assigned (by, e.g., verbally issuing the directive to patrol the area), a change of goal is detected and an episodic marker (Ep-1) is encoded, and linked with the encoded goal. As the agent proceeds through the task, it encounters new locations. For each change of location, a new episodic marker is encoded (Ep-2, Ep-3, Ep-4), and populated with details regarding the changes in location, as well as the prior episodes. At one point, the agent encounters a new individual (e.g., Bob). It encodes one episodic marker to capture Bob's arrival, and another to capture Bob's departure. Once the patrolling goal is accomplished, a new marker is encoded. In line with extant theories of event segmentation, the process of encoding events is continuous. As the agent moves on to other tasks, more episodic markers are created and stored in memory.

To perceive an event as an event, the system must retrieve the markers in memory and use them to retrospectively construct an event model. We turn to this procedure.

#### Event model construction

Event segmentation occurs on an ongoing basis by default, i.e., episodic markers are encoded online. In contrast, event models are only constructed retrospectively, as a result of an external query. It is from these models that people make inferences about temporal matters. For example, the user can query the system to remember a particular location, or to infer a particular relation that holds between events, or to describe the events that occurred in a given time window. Retrospective construction is highly relevant when the system needs to make inferences about its recent experiences. For example, if the system is directed to perform a particular goal—as in the patrol example above—then it will have two separate episodic markers that highlight the start of a new goal and its completion, along with any associated environmental information that the system can detect. Now suppose that during the course of the goal, the system traveled to two separate locations. That means that the system will construct at least four separate episodic markers:

1. A marker representing the start of a new goal.
2. A marker representing the detection of a new location (*location 1*) as well as the current goal.
3. A marker representing the detection of a new location (*location 2*) as well as the current goal.
4. A marker representing the satisfaction of the goal.

These four markers will be represented in long-term memory. When the system is prompted to recall information about the particular goal, it can retrieve all four markers. It parses markers (1) and (2) to build a model of a goal's duration:

goal_START_                                   goal_END_

Information provided from markers (2) and (3) allow for the construction of the durational event marking location 1:

$$\begin{array}{l} \begin{array}{cc} {\text{goal}}_{\text{START}} & \;{\text{goal}}_{\text{END}} \end{array}\\ \;{\text{location1}}_{\text{START }}{\text{location1}}_{\text{END}} \end{array}$$

and information provided from markers (3) and (4) allow for the construction of the durational event marking location 2:

$$\begin{array}{l} \begin{array}{cc} {\text{goal}}_{\text{START}} & \;{\text{goal}}_{\text{END}} \end{array}\\ \;{\text{location1}}_{\text{START }}{\text{location1}}_{\text{END}}\\ \;{\text{location2}}_{\text{START }}{\text{location2}}_{\text{END}} \end{array}$$

Hence, a complete event model of the relevant experiences is represented in the following mental model:

**Table:** 

goal_START_		goal_END_
	location1_START_ location1_END_	
	location2_START_	location2_END_

From the model above, individuals can draw deductions concerning event relations, such as that visiting location 1 occurred during the goal, and the visit to location 1 occurred before the visit to location 2. The model can be revised and modified, in which case inferences would be counterfactual (Byrne, 2005). For example, reasoners can modify the event model to move the duration of the visit to location 1 *after* the visit to location 2. If no other changes are made to the model, then the reasoner might make the following counterfactual conclusion: *if the visit to location 1 had happened after the visit to location 2, then it would not have happened while the system was completing the goal*. In sum, episodic chunks can be used to build complex event models from memories. Scanning and revising the models accordingly serves as the basis of temporal reasoning.

**Figure 4:**
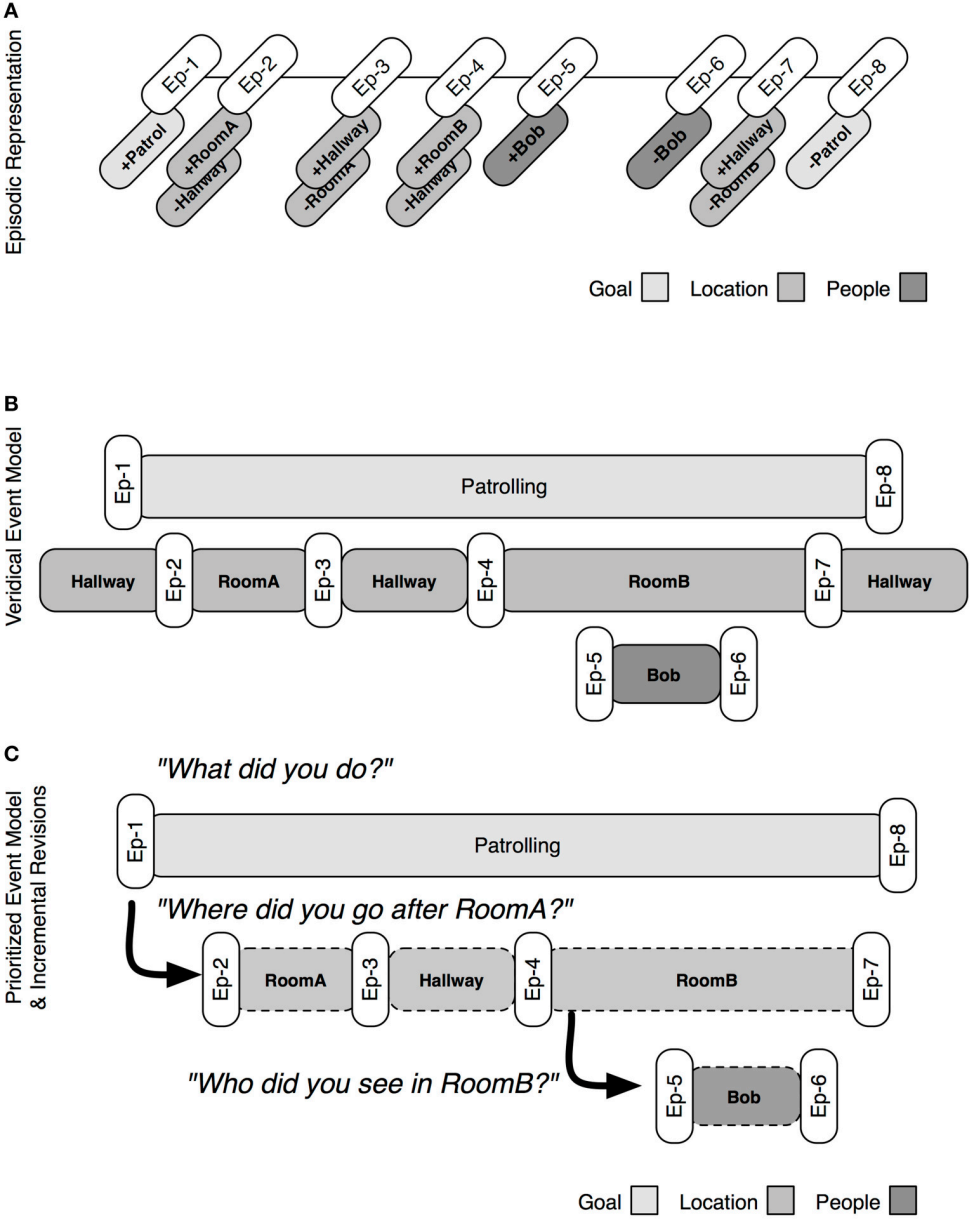
**The process by episodic markers are retrieved to build event models**. **(A)** Shows the episodic representation (see also Figure 3B). **(B)** Shows a veridical event model that can be constructed by an unprioritized mapping from episodic markers to model structures. **(C)** Shows a prioritized mapping, in which the construction of a goal event takes precedence to that of other sorts of events. Additional queries can be used to revise and flesh out the prioritized event model.

The basic process for constructing an event model is illustrated in Figure 4. At the top of the figure is the episodic representation that was built in the patrolling example above (Figure 3B). The system constructs an event model by retrieving the earliest relevant episodic marker (e.g., Ep-1) and checking how it was triggered (e.g., goal change). From this information, a provisional event encoding is created and associated with content regarding the type and trigger for the event (e.g., a goal change initiated by following a command to patrol a given area). This information is retained until a compatible episodic marker (e.g., Ep-8) is retrieved, marking the end of the event and committing it to the event model. Each episode is retrieved and processed until there are no more markers, or some temporal limit is reached.

The process is able to produce veridical event models, such as that seen in Figure 4B: a veridical event model is a one-to-one mapping of marker pairs and events. Humans are unlikely to generate such complex and complete event models, particularly over long periods of time. Instead event models are influenced by the goals that triggered the retrospective construction in the first place. The principle of event prioritization constrains the construction of episodic marker types. By default, this prioritization is (from highest to lowest priority): goal, location, person, and object. During reconstruction, lower prioritized events are only encoded when they fall within the bounds of higher prioritized events. In this way, an implicit sub-event model structure can be reconstructed. Figure 4C shows the prioritized event model, which only represents the superordinate event, i.e., the event that characterizes the goal of patrolling an area. The principle of event prioritization, while specifying a default prioritization, does not exclude the possibility that other retrospective tasks could require other prioritizations. User queries may demand some information over others and prioritize, e.g., locations to be retrieved. The system supports the construction of partial, incremental event models.

A demonstration of the system for event segmentation and model construction as it occurs online is available in the Video 1.

## General discussion

We describe a unified synthesis of event segmentation and temporal reasoning. Researchers typically focus on one process or the other. In our treatment, both are organized around the construction of discrete temporal mental models (i.e., event models). Models serve as the output of the event segmentation and the basis of temporal inference. Event segmentation is relevant in the online perception of events. Humans are capable of applying a regimented hierarchy to the continuous stream of sensory input they receive, and do so automatically and without difficulty. Yet no current theory of event segmentation or computer algorithm explains how different pieces of environmental input are used to regiment the stream of input. We accordingly developed an algorithm based on two overarching principles: (i) individuals represent events by constructing markers that track perceived changes in goals, locations, individuals, and objects; and (ii) episodic markers are constructed based on a prioritization hierarchy, in which changes in goals take precedence to changes in location, and changes in location take precedence to changes in characters and objects. The theory provides a plausible mechanism for temporal reasoning. The account thus unifies temporal cognition from how time is perceived to how temporal relations are inferred. The two principles upon which the account is based are simulated in a computational implementation of the theory, and on a robotic platform that demonstrates the viability of the hypotheses are guiding online perceptual input.

In addition to advancing temporal cognition, our theory is grounded in systematic evidence from cognitive neuroscience. The approach demonstrates a central role for neuroscientific research in the development of cognitive theory. We conclude by discussing a recent controversy on the role of cognitive neuroscience in developing and testing psychological theories of reasoning.

A central and irreproachable result from recent studies of the neuroscience of deductive inference may be that it is not modular: it implicates large swathes of the brain. A given experiment can show activation in various configurations of the basal ganglia, cerebellum, and occipital, parietal, temporal, and frontal lobes (Goel, 2007; Prado et al., 2011). Different sorts of inference recruit different brain regions (e.g., Waechter and Goel, 2005; Kroger et al., 2008; Monti et al., 2009), and a recent meta-analysis of 28 neuroimaging studies revealed systematic consistency in those regional activations for relational, quantificational, and sentential inferences (Prado et al., 2011).

Despite evidence of systematicity, many skeptics question if neuroimaging data can ever help adjudicate between theories of cognitive operations (Harley, 2004; Coltheart, 2006; Uttal, 2011). The problem is acute for students of reasoning: in order to make use of the available data, predictions of functional neuroanatomy are coaxed from psychological proposals. Most cognitive accounts of inference make no strong claims about functional neuroanatomy (Heit, 2015), i.e., they make no claims at the “implementation level” of inference (see Marr, 1982). Hence, coaxing predictions about implementation from accounts that specify only the mathematical functions to be computed for reasoning, or else the representations and algorithms that underlie reasoning, has the insidious effect of washing away theoretical nuances (Goel, 2007). Many imaging studies test the extreme view that the biological implementation of inferential procedures should rely on only one sort of mental representation, which has a distinct neural signature. The preponderance of evidence conflicts with such a view (Prado et al., 2011), which is fortunate, because the present authors know of no author or theory that defends it. And as Oaksford (2015) observes, constraints on the methodology itself may prevent diagnostic analyses. Researchers accordingly face a methodological quandary: Is it possible to marshal insights from cognitive neuroscience to inform theories of reasoning when those theories fail to make predictions of neural mechanism?

Our present approach demonstrates that it is indeed possible for theories of inferences to be informed by insights from cognitive neuroscience. As in previous work on developing an embodied theory of spatial cognition (Trafton and Harrison, 2011), we describe an embodied theory of temporal cognition whose fundamental assumptions are informed and constrained by recent work on the neuroscience of temporal processing. Cognitive neuroscience may be in its infancy, and likewise, theories of inference do not make predictions that can be tested by the imaging methodologies. Nevertheless, results from imaging studies rule out certain sorts of representations and provide mechanistic constraints on how humans may engage in particular cognitive tasks. The preceding discussion serves as a case study in how neuroimaging results can serve to guide and constrain the development of theories at Marr's “algorithmic level,” which focuses on cognitive representations and processes upon those representations.

In particular, the representations we proposed in the present theory—episodic markers and event models—are supported by work on how event segmentation is carried out by the brain. Likewise, the procedures we posit, including the hypothesis that people prioritize certain changes in the environment over others, are guided by both behavioral and imaging work on mental processes that track ongoing changes in the environment. Hence, cognitive neuroscience can play a pivotal role in the development and enrichment of cognitive theories of reasoning: imaging research can serve to rule out representations that cannot be feasibly processed by complementary neural processes, and it can suggest the need for alternative representations.

The skeptics may ultimately have purchase: no psychological theory of reasoning can be said to be testable by means of neuroscientific data unless that theory makes specific predictions of neural processes. A first step toward such a theory for any domain of cognition is to provide a unified account of that domain that explains how low-level perception leads to high-level inference. In the case of temporal cognition, we provide such an account, and explain how events are perceived to build mental simulations of their temporal experience, and how reasoners make temporal inferences from those simulations.

### Conflict of interest statement

The authors declare that the research was conducted in the absence of any commercial or financial relationships that could be construed as a potential conflict of interest.

## Supplementary material

Video 1 **A demonstration of the online process of perceptual segmentation, episodic marker encoding, and event model construction on an embodied robotic platform**.
